# Road Descriptors for Fast Global Localization on Rural Roads Using OpenStreetMap

**DOI:** 10.3390/s23187915

**Published:** 2023-09-15

**Authors:** Stephen Ninan, Sivakumar Rathinam

**Affiliations:** Texas A&M University, College Station, TX 77843, USA; srathinam@tamu.edu

**Keywords:** OpenStreetMap, localization, particle filter, road segmentation, descriptors

## Abstract

Accurate pose estimation is a fundamental ability that all mobile robots must posses in order to navigate a given environment. Much like a human, this ability is dependent on the robot’s understanding of a given scene. For autonomous vehicles (AVs), detailed 3D maps created beforehand are widely used to augment the perceptive abilities and estimate pose based on current sensor measurements. This approach, however, is less suited for rural communities that are sparsely connected and cover large areas. Topological maps such as OpenStreetMap have proven to be a useful alternative in these situations. However, vehicle localization using these maps is non-trivial, particularly for the global localization task, where the map spans large areas. To deal with this challenge, we propose road descriptors along with an initialization technique for localization that allows for fast global pose estimation. We test our algorithms on (real world) maps and benchmark them against other map-based localization as well as SLAM algorithms. Our results show that the proposed method can narrow down the pose to within 50 cm of the ground truth significantly faster than the state-of-the-art methods.

## 1. Introduction

Every Autonomous Vehicle needs to answer three fundamental questions—Where am I? Where am I going? and How do I get there? The process of answering the first question is what is commonly referred to as localization. To solve the localization problem means to estimate a robot’s pose in a predefined map of the environment. While most of the existing work is focused on solving the localization problem for autonomous vehicles (AVs) operating in urban areas, very little work has been performed for localization on rural roads—more specifically, for localization on rural roads using OpenSteetMaps. Within this paper, we present a refined methodology using OpenStreetMap for rural road localization using only visual sensors and odometry measurements.

In general, a vehicle’s ability to safely navigate a given environment is highly dependent on its understanding of the environment and its pose within the environment. An accurate and reliable pose estimate is therefore critical for the safe functioning of AVs. Although the accuracy of position sensors such as GPS has improved significantly over the past few years, GPS outages are still an area of concern, which can be catastrophic for an AV. In this article, we propose an initialization algorithm to deal with such a GPS-denied or a GPS-restricted environment.

A common approach [1] to solve the localization problem is through the use of pre-constructed environment maps, along with sensor and motion measurements. Depending on the sensors used by the vehicle, the map representation could be anything ranging from a simple position vector, containing positions of artifacts in the map frame, to a more complex representation, such as a dense 3D map containing point-wise annotations. As such, environment maps are useful not only for localization but also for other functions, such as motion and path planning, besides also improving robustness of the perception system.

In the context of self-driving vehicles, the map representation that is most often used is in the form of dense feature maps with detailed annotations for features such as lane markings and traffic signs. Although the use of such maps has been highly successful for structured environments such as urban roads, their advantage is less pronounced for rural driving scenarios because of three main reasons—structure, scale and sparsity of features—each of which is tied to the makeup of rural roads.

The first and most important distinction between urban and rural roads is their structure. While urban roads usually have a well-defined and consistent structure, rural roads can have large variations in structure, such as inconsistent road markings and varying road surfaces, such as gravel or dirt roads apart from the usual asphalt or concrete found on urban roads.

**Figure 1:**
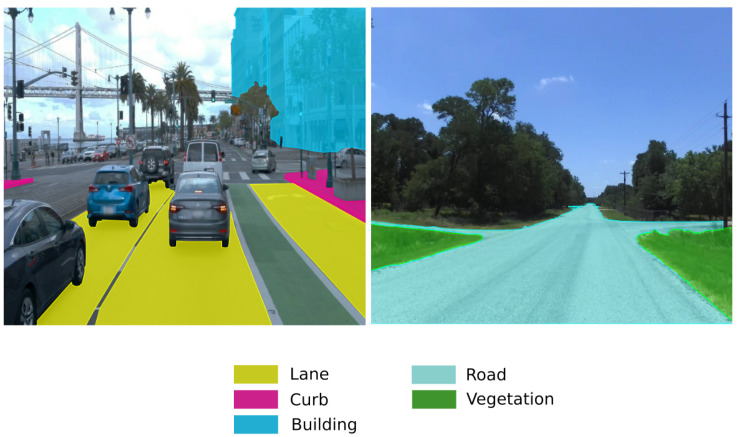
Comparison between urban and rural scenes.

Another problem with using dense maps for rural roads is their scale and sparsity of features. Urban scenes generally contain a rich feature space composed of features such as traffic signs, buildings, curb and lane markings, to name a few. Compared to urban communities, rural communities span very large areas and have very low population densities. Because of this, rural roads are primarily surrounded by vegetation and any features useful for localization are few and far between. An example of this structural difference is shown in Figure 1. Notice that the urban scene has several useful landmarks, such as buildings, sidewalks and lane markings. Our proposed method avoids these issues by leveraging the ubiquity of road geometry as a feature for localization. Our use of OpenStreetMaps also helps to deal with the large areas that rural communities tend to encompass, as there is no need to build detailed 3D maps.

Topological maps, such as OpenStreetMap, have been shown to be useful for autonomous driving scenarios, where the length of the trip is very large [2], or for locations where dense 3D maps are not available.

These maps, however, do not contain low-level features, such as lane markings and traffic signs. Pose estimation using these maps alone is therefore a significant challenge.

Our focus in this paper is on developing localization algorithms for rural roads. Our approach is useful even when GPS signals are disrupted due to jamming (intentionally or unintentionally) [3,4], or while traveling through mountainous regions or in scenarios with challenging weather conditions [5,6]. Within this work, we propose a novel road descriptor as a concise feature representation for rural roads. We use these road descriptors to generate an initial pose belief which is then passed to a particle filter for localization. The choice of the initial belief significantly impacts the rate of convergence of particle filter-based localization algorithms, especially for global localization, where the search space is very large. The road descriptor search (RDS) technique we introduce helps in the selection of this initial belief. We demonstrate this through simulations as well as real-world tests by comparing the performance of the localization algorithm with and without the generation of an initial belief. Results show that our RDS initialization significantly reduces the time to convergence for global localization compared to the state of the art. The algorithm is also able to estimate the pose of an ego vehicle in a map spanning 36 sq. km with a mean error of 1.5 m. Our software is open source and can be accessed here https://github.com/nsteve2407/osm-localization (accessed on 30 July 2023).

The rest of this paper is organized as follows: Section 2 contains a summary of the existing work on localization using topological maps. In Section 3, we introduce the road descriptor along with the Monte Carlo localization algorithm. Lastly, Section 4 contains results from both simulations and real-world tests to corroborate the effectiveness of our approach.

## 2. Related Work

The use of topological maps for localization has been widely researched as an alternative to traditional mapping. Hentschel et al. [7,8] used a Kalman filter for fusing noisy GPS observations with laser scans and a 2D line feature map that enabled localization near buildings. In OpenStreetSLAM [9], the authors propose a chamfer matching algorithm to estimate a vehicles pose by matching the vehicle’s trajectory over a period of time to a sequence of edges on the map.

A common trend among recent works in this area is the use of a particle filter for localization, with different measurement models proposed to correlate visual information with the information available in OpenStreetMap. Ruchti et al. [10] use road points from laser scans along with a zero mean Gaussian for the observation model; similarly, MapLite [11] proposes a signed distance function for the measurement model. Learning-based methods have also been proposed to learn the measurement function. One such work was conducted by Chen et al. [12], in which the authors train a model to embed road images to their corresponding map tiles, which is then used as a measurement model.

More recently, the idea of descriptors has been proposed as an alternative to previously cited distance-based measurement models. In [13], image-based oriented and rotated brief (ORB) descriptors are used to generate a bag of words, which is then queried for future measurements. A 4-bit descriptor encoding surrounding structural information, such as intersections and buildings, is introduced by Yan et al. [14]. This approach is further extended through the use of building descriptors as a unique feature vector for a pose on the map by Cho et al. [15]. This method measures the distance to the surrounding building walls at a given pose to form a rotation-invariant descriptor. Although this method is successful for urban routes, it is less suitable for rural scenarios, where building information is non-existent and most roads are open roads.

Since features such as buildings and landmarks are absent on rural roads, we propose a descriptor that utilizes a key feature that is consistent on all rural roads—*road geometry*. We do this by proposing road descriptors as a concise representation of road geometry for any given pose for rural roads. We then combine the advantages of descriptors as well as distance-based measurement functions for fast and accurate global pose estimation on rural roads.

## 3. Methodology

Localization can be mathematically defined as the task of calculating the belief of a robot’s pose, $bel(x_{t})$ at time *t*, by combining information from previous sensor measurements $z_{1:t}$, control inputs $u_{1:t}$, and map information *m*. In other words,

(1) $$bel\left(x_{t}\right)=p\left(x_{t}|z_{1:t},u_{1:t},m\right).$$

By applying Bayes rule, Equation (1) can be rewritten as

(2) $$p\left(x_{t}|z_{1:t},u_{1:t},m\right)=\frac{p\left(z_{t}|x_{t},z_{1:t-1},u_{1:t},m\right)p\left(x_{t}|z_{1:t-1},u_{1:t},m\right)}{p(z_{t}|z_{1:t-1},u_{1:t},m)}.$$

We can further simplify Equation (2) using a Markovian assumption and by applying the law of total probability to obtain a recursive function:

(3) $$p\left(x_{t}|z_{1:t},u_{1:t},m\right)=\frac{p\left(z_{t}|x_{t},m\right)\sum_{i}p\left(x_{t}|u_{t},x_{t-1}^{i}\right)bel\left(x_{t-1}^{i}\right)}{p(z_{t}|m)}.$$

Equation (3) is commonly referred to as the Bayes filter equation, which can be divided into three components: the motion model, which takes into account control input $u_{t}$ represented by $\sum_{i}p\left(x_{t}|u_{t},x_{t-1}^{i}\right)bel\left(x_{t-1}^{i}\right)$; the observation model, represented by $p(z_{t}|x_{t},m)$; and the normalizing factor, $p(z_{t}|m)$. This is to ensure that the total probability sums to one. Our implementation of the motion and observation models as well as the proposed road descriptors are discussed in the following subsections.

### 3.1. Road Descriptors and Motion Model

As seen in Equation (3), the motion model depends on the previous state $x_{t-1}$ and the input $u_{t}$. Since this is a recursive algorithm, the previous state needs to be initialized for the first iteration. The choice of the initial belief significantly impacts the rate of convergence of these Monte Carlo-style localization algorithms, especially for global localization tasks, where the search space is very large (we demonstrate this later in the Section 4). To narrow down the search space and generate a better initial belief, we propose road descriptors, which embed road geometry information at a given position on the map. For rural scenes, roads are the visual features of choice because unlike urban scenes, they most often do not contain other consistent features, such as buildings.

**Figure 2:**
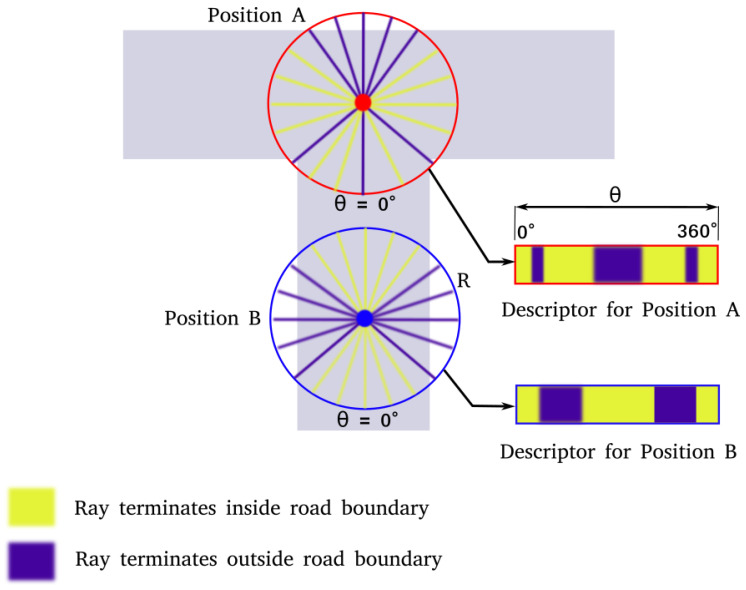
Generating road descriptors from OpenStreetMap.

The road descriptor for any point *p* on the map is a 2-dimensional binary array *D* with rows corresponding to the distances between *p* and the road features, and columns corresponding to the angle subtended by the position vectors of the road features with respect to *p*. The values in *D* are generated by a ray-casting operation as illustrated in Figure 2. To form one row of *D*, we project a ray of length *r*, radially outwards, starting at *p*. This is carried out for all angles θ in intervals of size 1 degree. The value is then assigned depending on whether the ray terminates at a road point or not, based on the following rule:

$$D\left(r,\theta \right)=\left\{\begin{array}{cc} 1, & \mathrm{if}\;\mathrm{the}\;\mathrm{ray}\;\mathrm{lands}\;\mathrm{on}\;a\;\mathrm{road}\;\mathrm{point}\;\\ 0, & \mathrm{otherwise} \end{array}\right.$$

**Figure 3:**
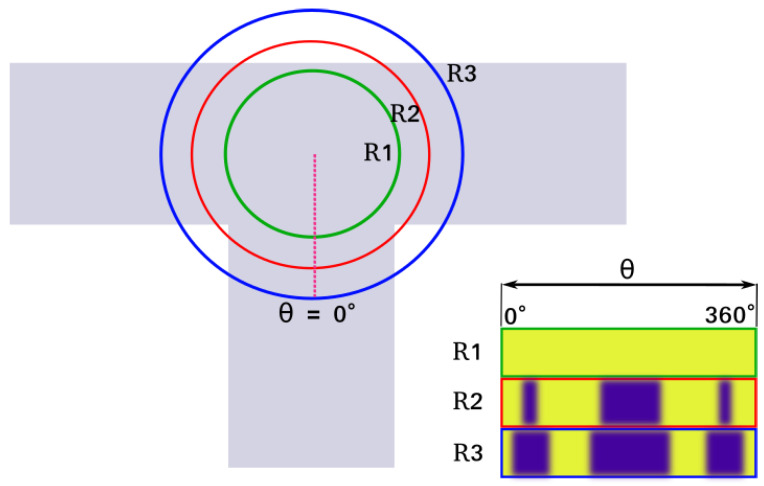
Concept of 2-dimensional road descriptors.

The result of this calculation for a given radius for two different positions on the road is shown in Figure 2. Repeating this operation for multiple radii, multiple rows can be generated, and the road descriptor for the point *p* can be computed as shown in Figure 3. In our framework, such road descriptors are precomputed for each node on OpenStreetMap and stored in a lookup table for the initialization step.

**Figure 4:**
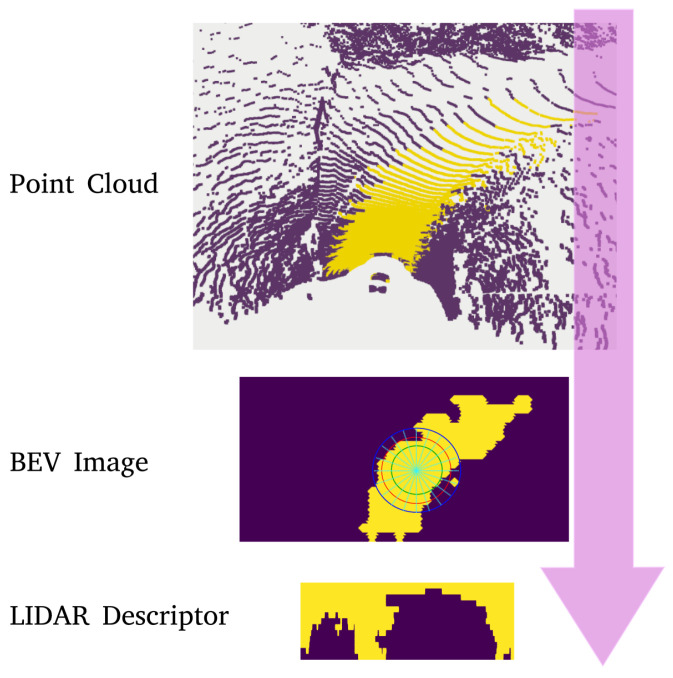
Generating road descriptors from segmented point clouds.

Measurements $z_{t}$ for the filter are LiDAR point clouds with labels for road points. In order to correlate point cloud data with road descriptors, point clouds are transformed into LiDAR descriptors to enable a descriptor search during the initialization phase. For this, the point clouds are first projected to a bird’s eye view (BEV) image as shown in Figure 4. The LiDAR descriptor *L* is then generated using the same ray-casting process explained for road descriptors. The ray casting in this case is performed starting from the center of the BEV image.

**Figure 5:**
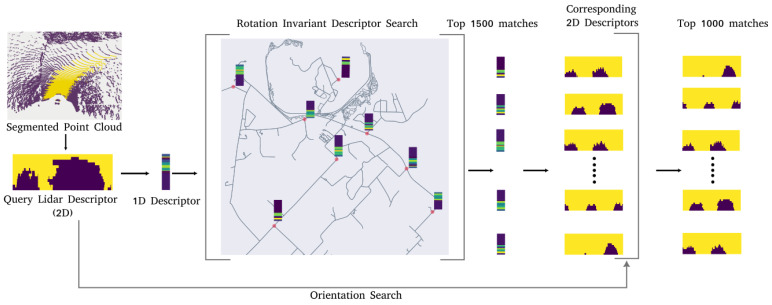
Descriptor search process.

Now, to generate the initial belief $bel(x_{t_{0}})$, the road descriptor search (RDS) process is used. The LiDAR point cloud is first converted into the descriptor form. This descriptor now acts as the query descriptor *L* for the search steps. RDS consists of two steps. In the first step, we only search the map for positions without accounting for orientation. This is because a change in the vehicle orientation would only result in a horizontal shift in the corresponding road descriptor values. We, therefore, make the road descriptor orientation invariant similar to [15]. We do this by computing the row sum for each row, and thus converting them into a 1-dimensional vector as shown in Figure 5. A search is then performed for the top 1500 positions on the map, where the 1-dimensional road descriptors are similar to the query descriptor.

To find the top 1500 similar descriptors *S* and their corresponding positions, a similarity score for a query (*L*) and road descriptor (*D*) pair is defined, which is

$$SimilarityScore=\frac{1}{||L-{D||}_{2}},$$

where ${\left||x|\right|}_{2}$ denotes the $L_{2}$-norm of *x*.

In the second step, to further reduce the number of matches for a given query descriptor, the orientation is also accounted for. The full 2-dimensional descriptors for *L* and *R* are used in this case. To do this, for each position in *S*, all orientations between 0 and 2π and their corresponding descriptors are considered. These are then sorted by their similarity score, and the top 1000 are selected as poses for initializing the particle filter.

For the motion model, we have the control input $u_{t}={\left[de,dn,d\theta \right]}^{T}$ which represents the estimated change in pose between time steps t−1 and *t*.

The control input $u_{t}$ along with initial estimates $x_{t-1}$ are used to generate a pose hypothesis ${\bar{x}}_{t}$ by sampling over the probability distribution as follows:

(4) $$p\left(x_{t}|x_{t-1},u_{t}\right)=N\left(x_{t}+u_{t},q\right)\;$$

where *q* represents the covariance of motion estimates.

### 3.2. Measurement Model

For the measurement model, a distance function is used to assign probability mass to each pose hypothesis ${\bar{x}}_{t}\in {\bar{X}}_{t}$. To calculate this distance function for a pose hypothesis ${\bar{x}}_{t}$, we transform the segmented point cloud to the map frame using the pose represented by ${\bar{x}}_{t}$, and based on the distance $d_{p_{road,i}}$ between the nearest road edge and a road point $p_{road,i}$ in the segmented cloud, we estimate $p(z_{t}|x_{t},m)$ as

(5) $$p\left(z_{t}|x_{t},m\right)=\prod_{i=1}^{1=n}\delta \left(d_{p_{road,i}}\right)\;$$

where *n* is the number of road points in the segmented point cloud, and δ represents the distance function, which is a Gaussian with zero mean and covariance *r* as proposed in [10]:

(6) $$\delta \left(d_{p_{road,i}}\right)=N\left(0,r\right).\;$$

For non-road points, $p_{non-road,i}$, an inverse probabilistic rule is used such that

(7) $$\delta \left(d_{p_{non-road,i}}\right)=1-\delta \left(d_{p_{road,i}}\right).\;$$

**Figure 6:**
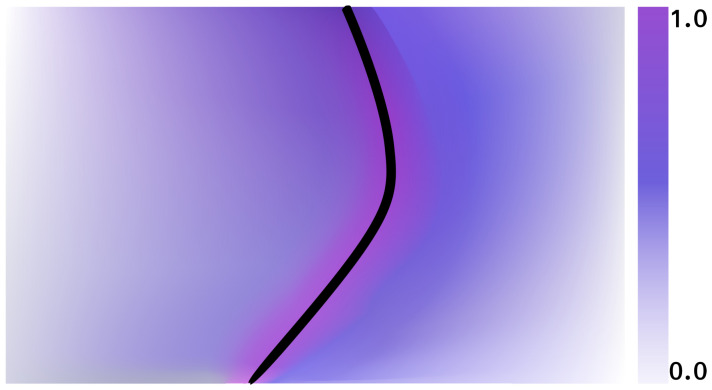
Returns from the distance function around a road edge on the map.

The distance function returns the maximum probability for projected points $p_{road}$ that are close to road edges on the map as illustrated in Figure 6. In effect, this means a greater probability mass will be assigned to poses where the road points $p_{road}$ are well aligned to the road geometry in the map. The probabilities of all pose hypotheses ${\bar{x}}_{t}\in {\bar{X}}_{t}$ are updated in this way. The poses are then sampled based on the assigned probability mass. Finally, the pose estimate $x_{t}$ is then estimated as the weighted average over all poses,

(8) $$x_{t}=\frac{\sum_{i}p\left(x_{i}\right)x_{i}}{\sum_{i}p\left(x_{i}\right)}\;$$

## 4. Experimentation and Results

To demonstrate the advantage of using road descriptors for the initialization of the prior belief, we perform two experiments. First, we simulate LiDAR road detection using OpenStreetMap. This is done to evaluate the road descriptor method independent of the performance of the road segmentation algorithm. Second, we evaluate the global localization performance of the Maplite algorithm [11] on real-world data, with and without the road descriptors, to corroborate the performance of the proposed approach.

### 4.1. Simulation Tests

For the simulation process, instead of using real-world point clouds with road labels, as explained in Section 3, we generate a virtual BEV image from OpenStreetMap. This image is simply a top–down snapshot of the portion of the map around the true pose $x_{t}$. The virtual BEV image is used to generate the LiDAR descriptor *L* as explained in Section 3.1. The LiDAR descriptor is then used as the query descriptor for the road descriptor search process.

**Figure 7:**
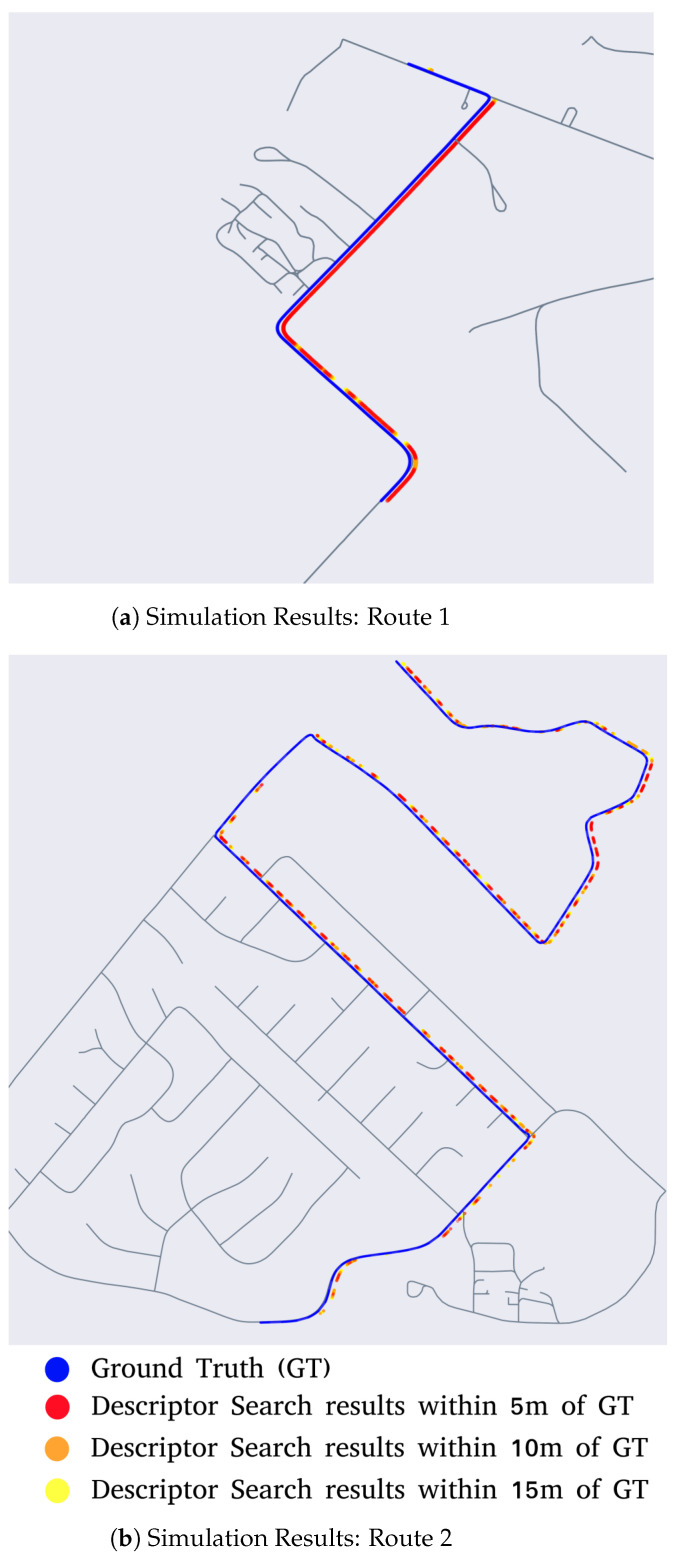
Results from LiDAR simulation tests.

In order to simulate a route, position and orientation information from OpenSteetMap nodes is used to generate ground truth measurements. For each ground truth measurement, the road descriptor search is performed over a segment of the map spanning 36 sq. km. The LiDAR descriptor generated from the virtual BEV image is used as the query descriptor. For each of the query descriptors, the top 1500 poses on the map that have similar descriptors are then found. If the true pose is within 5 m of the top 1500 matches, the descriptor search is considered to have converged. The results from this simulation are depicted in Figure 7a. The segments of the route where RDS has converged are highlighted in red, orange or yellow—based on the distance to ground truth. If RDS does not converge to within 15 m of the ground truth position, such nodes along the route are left uncolored.

From the simulation results, it can be seen that the descriptor search technique is most effective in segments of the route where there are road features such as turns and intersections, whereas it struggles in segments with only straight roads. This is prominent in the results from Route 2 (Figure 7b), where localization is lost in the straight portions of the route but subsequently regained at the portions with intersections and turns.

### 4.2. Real-World Tests

**Figure 8:**
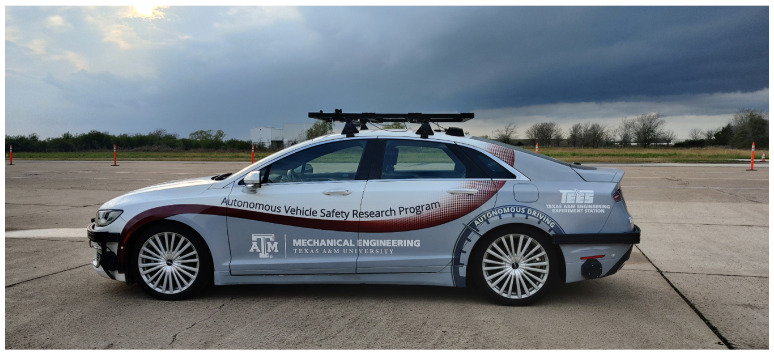
Vehicle platform used for real-time testing.

The performance of the particle filter with and without the proposed initialization using road descriptor search is evaluated by testing it on real-world data collected on two routes near Bryan, Texas. For data collection, a test vehicle (depicted in Figure 8) equipped with a 128 channel LiDAR sensor, a GNSS receiver with 2.0 m horizontal sensing accuracy, wheel speed and steering angle sensor is used. The measurements from the GNSS sensor are used only for evaluation and not used for the pose estimation algorithm.

**Figure 9:**
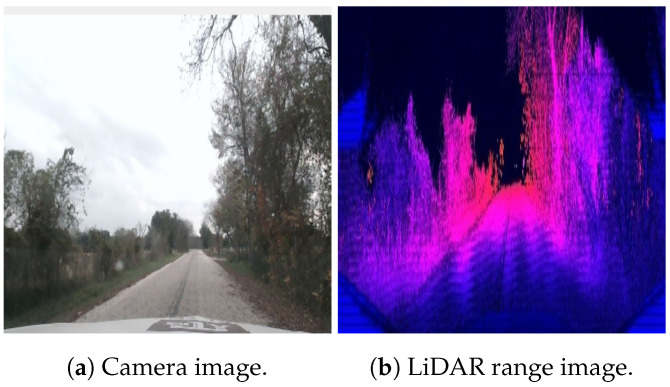
Example of data from the Texas A&M Autonomous Vehicle dataset.

To detect the road surface, a range image-based segmentation method based on RangeNet++ [16] is used. The model is trained on the Texas A&M Autonomous Vehicle rural road dataset [17]. The dataset contains 2800 range images (illustrated in Figure 9) labeled for the detection of road points. It also includes vehicle bus data, such as GPS, IMU, steering, brake, throttle inputs and wheel speed measurements. In all, the dataset contains around 15 min of driving data.

All the tests are run without using any of the GPS measurements. GPS logs are only used as ground truth for performance evaluation. The control input $u_{t}$ in Equation (4) is generated using a bicycle model, based on wheel speed and steering angle measurements. To reduce runtimes, we downsample the point clouds using a voxel grid, with voxels of size 2×2 m, and pre-compute the distance to the nearest edge for all points on the map. Similarly, road descriptors for all nodes on the map are pre-computed.

To demonstrate the computational advantages of the proposed approach, the size of the search space on the map is varied. For each of the routes considered, two tests are performed. For the first test, a search space of 9 sq. km is considered, followed by a 36 sq. km search space for the second test. For each of the tests, first the MapLite algorithm is initialized with 90,000 particles uniformly distributed over the entire search space. Next, MapLite is tested again but this time with RDS initialization. In this case, particles are initialized around the top 1500 matches returned by the descriptor search algorithm. Further validation is made by comparing the localization results after convergence with those obtained from SuMA SLAM [18].

**Figure 10:**
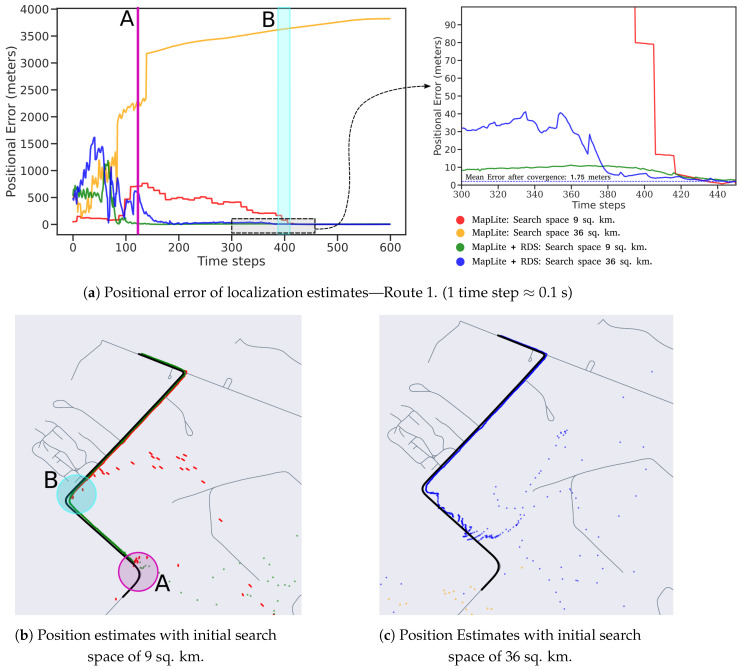
Localization Results for Route 1.

The first route represents data that were previously seen by the road segmentation model, as training data were collected on this route. This route contains dense vegetation on both sides and is therefore a challenging scenario for GPS-only or SLAM-only systems. An image from the route is shown in Figure 9. The localization results from this route are shown in Figure 10. It can be seen that the time taken by the Maplite algorithm to converge to the true position is significantly longer than when RDS is used for particle initialization.

When RDS initialization is used, a distinct reduction in time to convergence is observed both for the 9 and 36 sq. km search spaces. Referring to Figure 10b, for the 9 sq. km search test, the MapLite algorithm converges only after it sees road features up to turn B, which is indicated by the drop in error after around 400 time steps. In comparison, when RDS initialization is used, the algorithm quickly converges after around 100 time steps, when the vehicle reaches the turn ‘A’, and is therefore much faster than the earlier case.

Owing to the larger search space, the algorithm takes longer time to converge for the 36 sq. km search space. As seen in Figure 10c, when the algorithm is initialized without the descriptor search, it converges to an erroneous position on the map, which is indicated by the large position errors. This indicates that the number of particles used is inadequate with respect to the size of the search space. When road descriptors are used, the position estimate is close to the ground truth after the vehicle reaches turn A; however, there is still a small amount of error. This is because there are a few clusters of particles concentrated elsewhere on the map. These are subsequently ruled out after the vehicle reaches turn B, causing the algorithm to converge to the ground truth. The average position error (APE) after convergence for the proposed algorithm as well as the popular SLAM algorithm SuMA-SLAM [18] is reported in Table 1. We observe that the pose tracking performed by our algorithm is more accurate than the SLAM approach. This is primarily because of the lack of reliable feature points for SLAM, given that the route is mostly surrounded by vegetation. When noisy features, such as tree leaves, are used for pose estimation, the accuracy of the pose estimate between two scans decreases. These errors tend to accumulate over a period, tending to cause a drift in the pose estimates.

**Figure 11:**
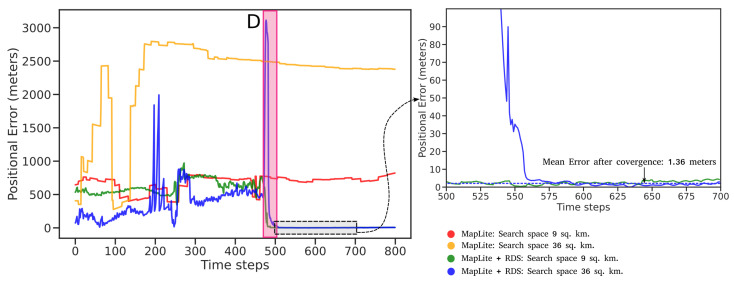
Positional error of localization estimates—Route 2. (1 time step ≈ 0.1 s).

**Figure 12:**
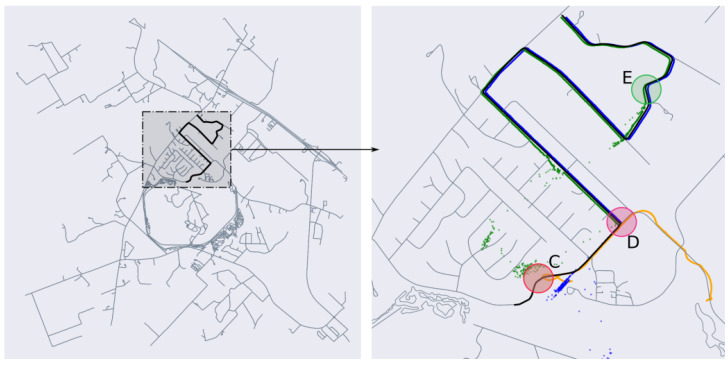
(**Left**) Search space spanning 36 sq. km, (**Right**) position estimates after convergence for Route 2.

Route 2 represents a more challenging test case because of the greater complexity and density of the road network in the region. There is also a greater diversity of road types, as parts of the route pass through rural residential areas. This is also a previously unseen route for the road segmentation model. Similar to Route 1, we perform the two tests on MapLite and MapLite + RDS algorithms, the results of which are shown in Figure 11 and Figure 12. It can be seen that MapLite alone fails to converge for both the 9 sq. km and 36 sq. km test cases, once again indicating that the number of particles was too small in comparison to the size and complexity of the map in consideration. It can be seen that when road descriptors are used for both these tests, the algorithm successfully converges to the true vehicle position. The large fluctuations in positional errors seen in Figure 11 in the initial phase is because of the presence of particles in other parts of the map that have a similar road geometry, such as turns C and E in Figure 12. However, when the vehicle reaches the intersection at D, those particle groups are eliminated, and the pose estimate thus converges to the ground truth after approximately 500 time steps. From Table 2, we observe once again that after convergence, the proposed algorithm has a lower APE than pose estimates from SLAM on the same route.

### 4.3. Runtimes

As seen from Route 2, the Maplite algorithm does not converge. This indicates that the number of particles is too low. However, with RDS initialization, the algorithm converges with the same number of particles. We compare the runtime of the Maplite algorithm with and without RDS initialization. Since Maplite alone does not converge with 90,000 particles, we increase the number of particles to provide a fair comparison with a configuration that converges to the true position with RDS. We also compare the runtimes with SuMA-SLAM [18]. Runtimes are reported in Table 3. It can been seen that runtimes are similar for the same number of particles. However, Maplite does not converge in this case. Even after increasing the number of particles to 150,000, the Maplite algorithm does not converge; however, the runtime almost doubles, thus making it unsuitable for real-time operation. This demonstrates the advantage of RDS, as it enables increasing the search space without increasing the number of particles needed for convergence.

### 4.4. Limitations

As mentioned previously, we use the top 1500 matches for initialization of the localization algorithm. In our tests, we find that for larger maps, if this number is reduced below 1000 matches, all particles very quickly converge to a single pose on the map, which may not necessarily be the right pose. This will cause all particle weights to drop to zero. We can use this to detect an erroneous localization and trigger RDS initialization again. The lower number of particles means that RDS initialization would be needed multiple times before the correct pose is recovered. Such a repeated initialization could also help deal with situations where the roads are mostly straight. Another limitation of our algorithm is the accuracy of OpenStreetMap. Since our algorithm is heavily reliant on road geometry from OpenStreetMap, good localization performance would depend on reasonably accurate data from these maps.

## 5. Conclusions

In this paper, we present a localization algorithm to enable global localization on rural roads. The proposed algorithm enhances the state of the art by using road descriptors for generating an initial belief. The algorithm is used to implement a LiDAR-based GPS-denied localization algorithm for global localization. Experimental results demonstrate that the algorithm can recover a vehicle’s pose, with a mean error as low as 0.5 m in maps as large as 36 sq. km. The performance enhancement provided by the RDS initialization makes this method suitable to deal with the kidnapping problem, especially for autonomous vehicles operating in regions with poor GPS reception.

## Figures and Tables

**Table 1:** Results after convergence: Route 1.

Method	Search Space	APE (m)	RMSE	σ
Maplite	9 sq. km	1.0	1.2	0.63
Maplite + RDS	9 sq. km	0.7	0.85	0.46
Maplite	36 sq. km	0.61	0.76	0.45
Maplite + RDS	36 sq. km	0.5	0.54	0.21
SuMA SLAM N/A	1.2	1.38	0.68	

**Table 2:** Results after convergence: Route 2.

Method	Search Space	APE (m)	RMSE	σ
Maplite	9 sq. km	-	-	-
Maplite + RDS	9 sq. km	0.81	0.7	0.45
Maplite	36 sq. km	-	-	-
Maplite + RDS	36 sq. km	0.81	1.08	0.7
SuMA SLAM	N/A	1.3	1.45	0.87

**Table 3:** Runtime comparison: Route 2.

Method	Particles	Runtime (ms)
Maplite	90,000	102
Maplite	150,000	192
Maplite + RDS	90,000	105
SuMA SLAM	N/A	120

## Data Availability

The data presented in this study are openly available in the VTTI Dataverse. https://doi.org/10.15787/VTT1/AOHI5N (accessed on 10 September 2023).
